# The collection of the Herpetological Museum of the University of Antioquia (northwestern Colombia)

**DOI:** 10.3897/BDJ.3.e1325

**Published:** 2015-02-05

**Authors:** Carlos E. Ortiz-Yusty, Juan M. Daza, Vivian P. Paez, Brian C Bock

**Affiliations:** † Grupo Herpetológico de Antioquia, Instituto de Biología, Universidad de Antioquia, Calle 67 # 53–108, Bloque 7–121, AA 1226, Medellín, Colombia

**Keywords:** Antioquia, tropical andes, neotropical, herpetology

## Abstract

Northwestern South America harbors one of the richest herpetofauna in the world. The connection among several biogeographic provinces along with climatic and orographic complexity makes this region an important contributor to the Neotropical biodiversity. Despite of this importance, the amphibian and reptile fauna in this area remains largely unknown as few herpetological collections has been made in recent decades. Motivated by this, the Herpetological Museum at the Universidad de Antioquia (Medellín, Colombia) has been increasing the collection in the last 16 years to better understand the herpetofaunal diversity and thus contribute to ecological, systematic, biogeographic and conservation research in the Neotropics. Here, we present the results of this effort and highlight how future collection will impact our understanding of the Neotropical herpetofauna.

## Introduction

The Herpetological Museum of the Universidad of Antioquia (in Spanish: Museo de Herpetología Universidad de Antioquia - MHUA) was created in 1998 with the main goal to generate, perpetuate, organize and provide information and data of amphibian and reptile faunas focusing mostly in northwestern Colombia in the Department of Antioquia and the northern Andes. The 13,343 specimens in the MHUA collection are mainly the result of the fieldwork and identifications of multiple researchers and students associated with the Biology Institute at the Universidad de Antioquia over the last sixteen years. Since its creation, the museum has been administered by the Antioquia Herpetological Group (in Spanish: Grupo Herpetológico de Antioquia - GHA, http://herpetologicodeantioquia.org/) and is currently directed by professors: Vivian P. Páez, Brian C. Bock and Juan M. Daza. Significant contributions to the growth, maintenance and optimal operation of the MHUA collection have also been made by past and current researchers: John J. Estrada, Juan M. Daza, Juan P. Hurtado, Esteban Alzate, Mauricio Rivera, Juan C. Arredondo, Eliana Muñoz, Lina Hinestroza, and Paul D. Gutiérrez-Cárdenas.

During the short history of the GHA and the MHUA, this collection has been a fundamental initiative, where specimens have been collected during research conducted by professors, biologists, and undergraduate and graduate students. It also has been essential for providing reference material for other studies conducted by the GHA research Lab and by other researchers from other national and international institutions. Of the current 13,343 specimens deposited in the MHUA collection, 8,195 are amphibians representing 230 identified species (~29.9% of the species in Colombia) and 5,148 are reptiles representing 223 identified species (~37% of the species in Colombia). Catalog numbers for amphibians range from MHUA A-0001 to MHUA A-8195 and for reptiles from MHUA R-10000 to MHUA R-18540. Specimens data are stored in a digital database including in some cases calls and pictures of the living specimen. Additionally, the museum houses seven holotypes and 142 paratypes of 11 species Table 1) and 240 tadpole lots preserved in formaldehyde and their data recorded on paper (Currently not included in the online database).

## Sampling methods

### Study extent

**Figure 1.**
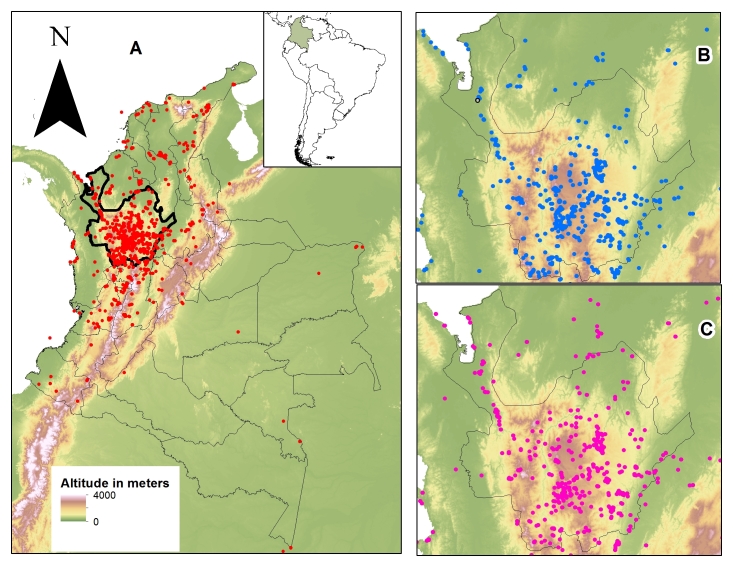
Geographic distributions of specimens deposited in MHUA collection. A: All specimens, Amphibian and Reptiles; B: Amphibians and C: Reptiles collected within Antioquia Province.

The MHUA is a regional museum covering mostly the northwestern region of Colombia (Fig. 1). Department of Antioquia with an area of 63,326.5 km^2^ is the most significant political division represented in the MHUA collection with 1,033 sampling localities and 8,544 specimens. Collection effort per locality varies from 1 up to 318 specimens.

### Sampling description

Specimens deposited in the MHUA are the result of multiple research projects, teaching activities, and donations. As a consequence, no general, single or uniform protocol has been followed to collect and prepare specimens. For example, collections procedures of donated specimens are largely unknown as most of the specimens were found fortuitously. In contrast, specimens from research projects and teaching activities share similar sampling and preparation protocols. Depending on the research goal, capture methods include Visual Encounter Surveys (VES), sampling plots, linear transects, and pitfall traps according to Angulo et al. (2006). All specimens are preserved in wet, alcohol for adults and formaldehyde for tadpoles.

### Quality control

Before a specimen is deposited in the collection, the museum curatorial staff revises the associated information to the specimen, i.e., locality, coordinates, taxonomy. The minimum information required to include a specimen in the collection is the one needed to publish the data in GBIF. Scientific names follow two standarized taxonomic nomenclatures. For amphibians we follow Frost (2014) and reptile taxonomy follows Uetz (2010).

## Geographic coverage

### Description

The 13,343 specimens deposited in MHUA collection came mainly from the Andean region of northwestern Colombia (Fig. 1 - A). Specimens have been collected from 1524 localities (953 for amphibians and 762 for reptiles), and the maximum number of amphibians (72.7% - 5959 specimens) and reptiles (53.1% - 2757 specimens) has been collected within province of Antioquia (1033 localities) (Fig. 1 - B:C). Antioquia has an area of 62 150 km2, which extends from the Atrato River on the west to the Magdalena River on the east, with a coastline of 240 km on the Gulf of Urabá on the Caribbean coast and two parallel mountain ranges (Cordillera Occidental and Cordillera Oriental) that bisect the department, attaining elevations up to 4 000 m.

**Figure 2.**
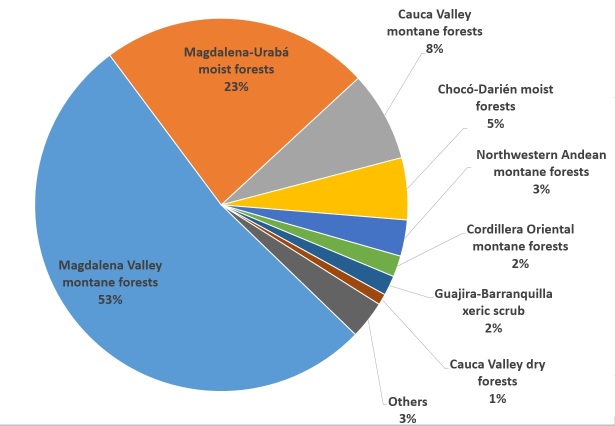
Biomes represented in MHUA collection (Suppl. material 1).

**Figure 3.**
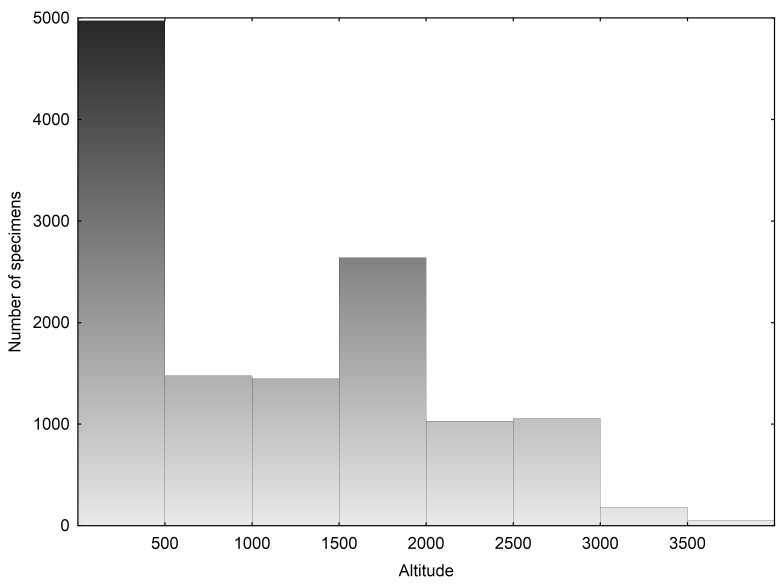
Altitudinal distribution of specimens in MHUA collections (Suppl. material 2).

MHUA contains specimens from 26 provinces within Colombia: Amazonas, Antioquia, Atlantico, Atlántico, Bolivar, Bolívar, Boyaca, Boyacá, Caldas, Caquetá, Cauca, Cesar, Choco, Chocó, Cordoba, Córdoba, Cundinamarca, Guajira, La Guajira, Loreto, Magdalena, Meta, Nariño, Norte de Santander, Quindío, Risaralda, San Andres y Providencia, Santander, Sucre, Tolima, Valle del Cauca, Vaupes and Vichada (Fig. 1). The biomes better represented in MHUA corresponds to Magdalena Valley montane forests with 6533 specimens (4749 amphibians and 1784 reptiles) and Magdalena-Urabá moist forests with 3029 specimens (1179 amphibians and 1850 reptiles; Fig. 2). Altitudinally, specimens in MHUA represents since sea level up to 4000 m. a. s., however, the majority of collections have been made in low and mid lands (below 2000 m a. s.; Fig. 3).

### Coordinates

4°20'9.6''S and 11°3'18''N Latitude; 78°12'0''W and 67°29'20.4''W Longitude.

## Taxonomic coverage

### Description

**Kingdom:** Animalia

**Phylum:** Chordata

**Classes:** Amphibia, Reptilia

**Orders:** Anura, Caudata, Gymnophiona, Crocodylia, Sauria, Serpentes, Squamata, Testudines

### Taxa included

**Table:** 

Rank	Scientific Name	Common Name
kingdom	Animalia	Animals
subkingdom	Eumetazoa	
phylum	Chordata	
subphylum	Vertebrata	
class	Amphibia	Amphibians
class	Reptilia	Reptiles
order	Crocodylia	
order	Sauria	
order	Serpentes	
order	Squamata	
order	Testudines	
order	Anura	
order	Caudata	
order	Gymnophiona	
family	Aromobatidae	
family	Brachycephalidae	
family	Bufonidae	
family	Caeciliidae	
family	Centrolenidae	
family	Ceratophryidae	
family	Craugastoridae	
family	Dendrobatidae	
family	Eleutherodactylidae	
family	Hemiphractidae	
family	Hylidae	
family	Leiuperidae	
family	Leptodactylidae	
family	Microhylidae	
family	Plethodontidae	
family	Ranidae	
family	Strabomantidae	
family	Plethodontidae	
family	Caeciliidae	
family	Rhinatrematidae	
family	Alligatoridae	
family	Amphisbaenidae	
family	Anguidae	
family	Corytophanidae	
family	Dactyloidae	
family	Gekkonidae	
family	Gymnophthalmidae	
family	Hoplocercidae	
family	Iguanidae	
family	Phyllodactylidae	
family	Polychrotidae	
family	Scincidae	
family	Sphaerodactylidae	
family	Teiidae	
family	Tropiduridae	
family	Anomalepididae	
family	Boidae	
family	Colubridae	
family	Dipsadidae	
family	Elapidae	
family	Leptotyphlopidae	
family	Tropidophiidae	
family	Typhlopidae	
family	Viperidae	
family	Colubridae	
family	Dactyloidae	
family	Dipsadidae	
family	Gekkonidae	
family	Gymnophthalmidae	
family	Iguanidae	
family	Scincidae	
family	Sphaerodactylidae	
family	Teiidae	
family	Viperidae	
family	Chelonidae	
family	Chelydridae	
family	Dermochelidae	
family	Emydidae	
family	Geoemydidae	
family	Kinosternidae	
family	Podocnemididae	

## Temporal coverage

### Notes

**Figure 4.**
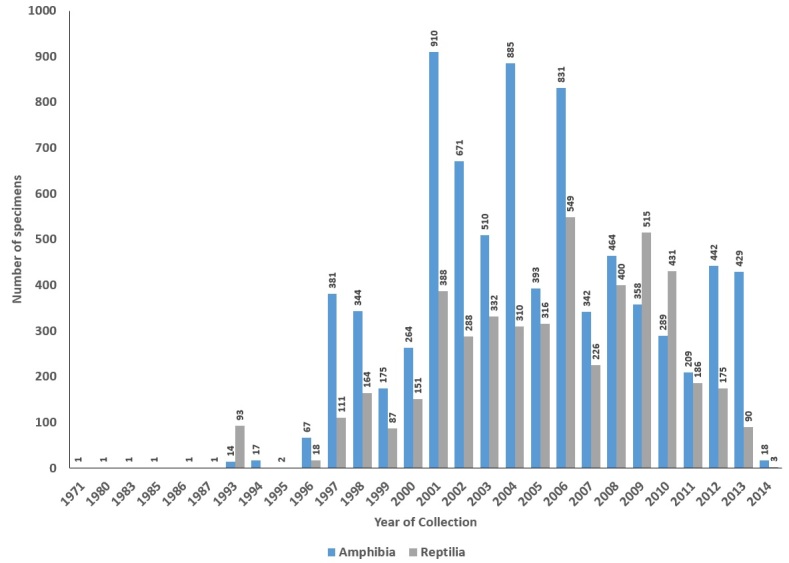
Temporal distribution of the specimen records in the MHUA collections (Suppl. material 3).

The MHUA data cover specimens collected from 1971 to 2013. Specimens collected before 1998, when the museum was founded, are mostly donations and sampling efforts of independent researchers. Most specimens have entered the collection after the year 2000 thanks to research projects in our laboratory and teaching activities (Fig. 4).

## Collection data

### Collection name

Museo de Herpetología Universidad de Antioquia

### Collection identifier

MHUA

### Specimen preservation method

Ethanol 70%

## Usage rights

### Use license

Creative Commons CCZero

## Data resources

### Data package title

Museo de Herpetología Universidad de Antioquia

### Number of data sets

2

### Data set 1.

#### Data set name

Colección de anfibios - Museo de Herpetología de la Universidad de Antioquia

#### Data format

Darwin Core Archive

#### Number of columns

35

#### Character set

UTF-8

#### Download URL

http://www.gbif.org/dataset/cc28549b-467f-448c-875e-881ca507aba8

#### Data format version

1.0

#### Description

**Data set 1.** 

Column label	Column description
occurrenceID	A unique identifier for the record within the data set or collection.
type	The nature or genre of the resource
language	A language of the resource
institutionID	An identifier for the collection or dataset from which the record was derived
institutionCode	The name (or acronym) in use by the institution having custody of the object(s) or information referred to in the record
collectionCode	The name, acronym, coden, or initialism identifying the collection or data set from which the record was derived
basisOfRecord	The specific nature of the data record
catalogNumber	An identifier for the record within the data set
recordedBy	A list of names of people, groups, or organizations responsible for recording the original Occurrence
preparations	A list of preparations and preservation methods for a specimen
eventDate	The date-time (year) during which an collection occurred
fieldNumber	An identifier given to the event in the field
continent	The name of the continent in which the Location occurs
country	The name of the country or major administrative unit in which the Location occurs
countryCode	The standard code for the country in which the Location occurs
stateProvince	The name of the next smaller administrative region than country in which the Location occurs
county	The full, unabbreviated name of the next smaller administrative region than stateProvince in which the Location occurs
locality	The specific description of the place. Less specific geographic information can be provided in other geographic terms
minimumElevationInMeters	The lower limit of the range of elevation (altitude, above sea level), in meters
maximumElevationInMeters	The upper limit of the range of elevation (altitude, above sea level), in meters
decimalLatitude	The geographic latitude (in decimal degrees, using the spatial reference system given in geodeticDatum) of the geographic center of a Location. Positive values are north of the Equator, negative values are south of it. Legal values lie between -90 and 90, inclusive
decimalLongitude	The geographic longitude (in decimal degrees, using the spatial reference system given in geodeticDatum) of the geographic center of a Location. Positive values are east of the Greenwich Meridian, negative values are west of it. Legal values lie between -180 and 180, inclusive
geodeticDatum	The ellipsoid, geodetic datum, or spatial reference system (SRS) upon which the geographic coordinates given in decimalLatitude and decimalLongitude as based
georeferenceRemarks	Notes or comments about the spatial description determination, explaining assumptions made in addition or opposition to the those formalized in the method referred to in georeferenceProtocol
identificationQualifier	A brief phrase or a standard term ("cf.", "aff.") to express the determiner's doubts about the Identification
scientificName	The full scientific name, with authorship and date information if known
kingdom	The full scientific name of the kingdom in which the taxon is classified
phylum	The full scientific name of the phylum or division in which the taxon is classified
class	The full scientific name of the class in which the taxon is classified
order	The full scientific name of the order in which the taxon is classified
family	The full scientific name of the family in which the taxon is classified
genus	The full scientific name of the genus in which the taxon is classified
specificEpithet	The name of the first or species epithet of the scientificName
taxonRank	The taxonomic rank of the most specific name in the scientificName
collectionID	An identifier for the institution having custody of the object(s) or information referred to in the record

### Data set 2.

#### Data set name

Colección de reptiles - Museo de Herpetología de la Universidad de Antioquia

#### Data format

Darwin Core Archive

#### Number of columns

35

#### Character set

UTF-8

#### Download URL

http://www.gbif.org/dataset/d3ba75d5-1382-440f-8f79-c45d8e3e69be

#### Data format version

1.0

#### Description

**Data set 2.** 

Column label	Column description
occurrenceID	A unique identifier for the record within the data set or collection
type	The nature or genre of the resource
language	A language of the resource
institutionID	An identifier for the collection or dataset from which the record was derived
institutionCode	The name (or acronym) in use by the institution having custody of the object(s) or information referred to in the record
collectionCode	The name, acronym, coden, or initialism identifying the collection or data set from which the record was derived
basisOfRecord	The specific nature of the data record
catalogNumber	An identifier for the record within the data set
recordedBy	A list of names of people, groups, or organizations responsible for recording the original Occurrence
preparations	A list of preparations and preservation methods for a specimen
eventDate	The date-time (year) during which an collection occurred
fieldNumber	An identifier given to the event in the field
continent	The name of the continent in which the Location occurs
country	The name of the country or major administrative unit in which the Location occurs
countryCode	The standard code for the country in which the Location occurs
stateProvince	The name of the next smaller administrative region than country in which the Location occurs
county	The full, unabbreviated name of the next smaller administrative region than stateProvince in which the Location occurs
locality	The specific description of the place. Less specific geographic information can be provided in other geographic terms
minimumElevationInMeters	The lower limit of the range of elevation (altitude, above sea level), in meters
maximumElevationInMeters	The upper limit of the range of elevation (altitude, above sea level), in meters
decimalLatitude	The geographic latitude (in decimal degrees, using the spatial reference system given in geodeticDatum) of the geographic center of a Location. Positive values are north of the Equator, negative values are south of it. Legal values lie between -90 and 90, inclusive
decimalLongitude	The geographic longitude (in decimal degrees, using the spatial reference system given in geodeticDatum) of the geographic center of a Location. Positive values are east of the Greenwich Meridian, negative values are west of it. Legal values lie between -180 and 180, inclusive
geodeticDatum	The ellipsoid, geodetic datum, or spatial reference system (SRS) upon which the geographic coordinates given in decimalLatitude and decimalLongitude as based
georeferenceRemarks	Notes or comments about the spatial description determination, explaining assumptions made in addition or opposition to the those formalized in the method referred to in georeferenceProtocol
identificationQualifier	A brief phrase or a standard term ("cf.", "aff.") to express the determiner's doubts about the Identification
scientificName	The full scientific name, with authorship and date information if known
kingdom	The full scientific name of the kingdom in which the taxon is classified
phylum	The full scientific name of the phylum or division in which the taxon is classified
class	The full scientific name of the class in which the taxon is classified
order	The full scientific name of the order in which the taxon is classified
family	The full scientific name of the family in which the taxon is classified
genus	The full scientific name of the genus in which the taxon is classified
specificEpithet	The name of the first or species epithet of the scientificName
taxonRank	The taxonomic rank of the most specific name in the scientificName
collectionID	An identifier for the institution having custody of the object(s) or information referred to in the record

## Supplementary Material

Supplementary material 1 Biomes represented in MHUA collection Data type: Microsoft Excel file
File: oo_8510.xlsx Carlos Ortiz-Yusty

Supplementary material 2 Altitudinal distribution of specimens in MHUA. Data type: Microsoft Excel file
File: oo_8511.xlsx Carlos Ortiz-Yusty

Supplementary material 3 Temporal distribution of the specimen records in the MHUA collections Data type: Microsoft Excel file
File: oo_8513.xlsx Carlos Ortiz-Yusty

## Figures and Tables

**Table 1.** Holotypes and Paratypes conserved in the MHUA Herpetological Collection

**Holotypes**	**Catalog Number**	**Reference**
*Dendropsophus norandinus*	MHUA-A 3781	Rivera-Correa and Gutiérrez-Cárdenas 2012
*Dendropsophus manonegra*	MHUA-A 7336	Rivera-Correa and Orrico 2013
*Atelopus nocturnus*	MHUA-A 5280	Bravo-Valencia and Rivera-Correa 2011
*Agalychnis terranova*	MHUA-A 7316	Rivera-Correa 2013
*Hyloscirtus antioquia*	MHUA-A 7227	Rivera-Correa and Faivovich 2013
*Anadia antioquensis*	MHUA-R 10537	Arredondo 2013
*Anolis anoriensis*	MHUA-R 11719	Velasco et al. 2010
*Pristimantis jaguensis*	MHUA-A 7239	Rivera-Prieto et al. 2014
**Paratypes**		
*Pristimantis batrachites*	MHUA-A 1574-1604, 1775-1780, 2023	Lynch 2003
*Dendropsophus norandinus*	MHUA-A 3768-3780, 3782-3790, 4092, 5274	Rivera-Correa and Gutiérrez-Cárdenas 2012
*Dendropsophus manonegra*	MHUA-A 7337, 7668	Rivera-Correa and Orrico 2013
*Atelopus nocturnus*	MHUA-A 2472, 5279, 5281-5286	Bravo-Valencia and Rivera-Correa 2011
*Agalychnis terranova*	MHUA-A 7317-7320	Rivera-Correa 2013
*Hyloscirtus antioquia*	MHUA-A 1707-1709, 1716, 2645-2647, 4140, 5708-5708, 6012, 6014, 6138-6139, 7228-7232, 7449-7453, 7568	Rivera-Correa and Faivovich 2013
*Allobates niputidea*	MHUA-A 1830, 1831, 2070, 2072, 2147-2151, 2515	Grant et al. 2007
*Anadia antioquensis*	MHUA-R 11254	Arredondo 2013
*Anolis anoriensis*	MHUA-R 11263, 11284, 11720-11728	Velasco et al. 2010
*Atractus paisa*	MHUA-R 14222, 14226, 14235-14254, 14261, 14274	Passos et al. 2009
*Pristimantis jaguensis*	MHUA-A 7240, 7241, 7247-7257	Rivera-Prieto et al. 2014
